# Evaluation of antigen-specific immune responses for leprosy diagnosis in a hyperendemic area in China

**DOI:** 10.1371/journal.pntd.0006777

**Published:** 2018-09-24

**Authors:** Xiaohua Chen, Yuan-Gang You, You-Hua Yuan, Lian Chao Yuan, Ying Zhang, Wen Yan

**Affiliations:** 1 Beijing Tropical Medicine Research Institute, Beijing Friendship Hospital, Capital Medical University, Beijing, China; 2 Beijing Key Laboratory for Research on Prevention and Treatment of Tropical Diseases, Capital Medical University, Beijing, China; 3 Henan Provincial People's Hospital, Zhengzhou, China; 4 Department of Molecular Microbiology and Immunology, Bloomberg School of Public Health, Johns Hopkins University, Baltimore, MD, United States of America; Insitut Pasteur de Tunis, TUNISIA

## Abstract

**Objective:**

To evaluate antigen-specific immune responses for leprosy diagnosis in a hyperendemic area in China.

**Methods:**

Eighty-three leprosy patients and 161 non-leprosy controls were enrolled from Hani-yi Autonomous Prefecture of Honghe, Yunnan Province, China. Leprosy patients were divided into multibacillary (MB, n = 38), paucibacillary (PB, n = 23), and post-multi-drug therapy (MDT, n = 22) groups. Controls were divided into the following groups: healthy household contacts (HHC, n = 119), tuberculosis (TB, n = 11), and endemic controls (EC, n = 31). The NDO-LID Rapid Test, *M*. *leprae* antigen-specific ELISA and antigen-specific IFN-γ secretion in a whole blood assay (WBA) were used to evaluate these subjects.

**Results:**

The NDO-LID Rapid Test achieved higher positive response rates in MB than in PB patients[94.7%(36/38) *vs* 65.2%(15/23)], and these rates were higher than those observed by ELISA using anti-LID-1[92.1%(35/38) *vs* 52.2%(12/23)], anti-NDO-LID[92.1%(35/38) *vs* 47.8% (11/23)], and anti-ND-O-BSA[89.5%(34/38) *vs* 60.9%(14/23)]. However, the NDO-LID Rapid Test also showed a higher positive response rate in the EC group (33.3%,10/31), which was higher than the rates observed for anti-NDO-LID (12.9%,4/31) and anti-ND-O-BSA (16.1%,5/31). *M*. *leprae* antigen-specific ELISA demonstrated relatively high specificity (86.84–97.37%) but low sensitivity (15.97–72.73%) in discriminating between leprosy patients and non-leprosy controls by ROC curve analysis. In contrast, *M*. *leprae* antigen-specific IFN-γ secretion detection achieved higher positive response rates in PB than in MB patients (positive ratio of MB *vs* PB: 40% *vs* 56% for LID-1, 28.6% *vs* 47.8% for ML89, 31.4% *vs* 60.7% for ML2044, and 31.4 *vs* 47.8% for ML2028) and could distinguish MB from EC when stimulated with ML89(AUC = 0.6664) and PB fromTB when stimulated with ML2044 and ML2028(AUC = 0.7549 and 0.7372, respectively).

**Conclusion:**

The NDO-LID Rapid Test and *M*. *leprae* antigen-specific ELISA are useful tools to assist in the diagnosis of leprosy patients, especially MB patients, although the former had higher sensitivity but lower specificity than the latter. *M*. *leprae* antigen-specific IFN-γ release assessed by WBA has diagnostic value for distinguishing PB from TB but not for distinguishing PB from HHC or EC. Screening novel *M*. *leprae-*specific antigens, combining different *M*. *leprae* antigens and a multi-cytokine analyte model may be needed for more effective diagnosis of leprosy.

## Author summary

Although the implementation of World Health Organization (WHO) multidrug therapy (MDT) treatment has drastically reduced the number of registered leprosy cases, new case detection rates have stabilized over the last decade, and leprosy remains an important health problem in many regions. Antigen-specific immune diagnostic tools are helpful for leprosy diagnosis but require broad evaluation in different populations from areas with hyperendemic leprosy. The NDO-LID Rapid Test, *M*. *leprae* antigen-specific ELISA and antigen-specific secretion of IFN-γ in a whole blood assay (WBA) can be used to diagnose multibacillary (MB) and paucibacillary (PB) leprosy patients. The authors found that in Honghe Autonomous Prefecture,Yunnan Province, China, the NDO-LID Rapid Test and *M*. *leprae* antigen-specific ELISA have the potential to be used as tools to assist in the diagnosis of patients with MB leprosy. The NDO-LID Rapid Test has higher sensitivity but lower specificity than the *M*. *leprae* antigen-specific ELISA. *M*. *leprae* antigen-specific IFN-γ secretion in WBA exhibited diagnostic value for distinguishing PB from TB but not for distinguishing PB from HHC or EC. This study provides an evaluation of antigen-specific immune responses for leprosy diagnosis in a hyperendemic area in China.

## Introduction

Leprosy, a chronic disease caused by *Mycobacteriumleprae (M. leprae)* infection, has a wide range of clinical outcomes correlated with the host's immune response to the bacilli[1,2].

Current leprosy control strategies rely on diagnosing the disease as early as possible, followed by prompt treatment with multi-drug therapy (MDT)[1]. The implementation of World Health Organization (WHO) MDT for widespread, worldwide treatment has drastically reduced registered leprosy cases from the approximately 12 million reported in 1985 to fewer than 250,000 reported in 2006[3]. Currently, leprosy is mainly diagnosed by clinicians using defined criteria, slit-skin smears and biopsies[4]. However, as the prevalence of the disease decreases, clinical expertise is diminishing, leading to extended delays between the onset of clinical signs and the diagnosis and consequent sustained transmission of *M*. *leprae*[5].

Leprosy patients are predominantly diagnosed by the appearance of disease signs, but they can also be characterized by the physical and histological attributes of skin or nerve lesions or by their immune response to crude or recombinant *M*. *leprae* antigens[6, 7, 8, 9]. It has been demonstrated that the immune response to crude or recombinant *M*. *leprae* antigens is helpful for detecting multibacillary (MB) leprosy patients by their antibody response[6], for the diagnosis of paucibacillary (PB) patients by antigen-specific CMI[7], and for monitoring the effectiveness of MDT in MB and PB leprosy patients by the antibody response and antigen-specific CMI, respectively[8]. The *M*. *leprae* antigens used for ELISA in this study were Leprosy IDRI diagnostic-1 (LID-1), a fusion protein developed by fusing the ML0405 and ML2331 genes[9, 10];NDO-LID, a conjugate of LID-1 with natural octyl disaccharide (NDO)[11];and ND-O-BSA, a synthetic PGL-I derivative. The NDO-LID Rapid Test in lateral flow-based format has been developed using NDO-LID. The single tetravalent 89-kDa fusion protein(ML89), designated LEP-F1, consists of the ML2028, ML2055 and ML2380 antigens[12]. A list of accession numbers/ID numbers for genes and proteins included in the NCBI search and mentioned in the text is shown inTable 1.

**Table 1:** List of accession numbers/ID numbers for genes and proteins included in the NCBI search and mentioned in the text.

Name	Gene ID	Description	Location	Aliases
ML0405	ID: 909138	hypothetical protein [*Mycobacterium leprae TN*]	NC_002677.1 (503217..504401)	ML0405
ML2331	ID: 908688	hypothetical protein [*Mycobacterium leprae TN*]	NC_002677.1 (2761703..2762473)	ML2331
ML2044	ID: 909000	hypothetical protein [*Mycobacterium leprae TN*]	NC_002677.1 (2434368..2434589, complement)	ML2044
ML2028	ID: 909036	fbpB diacylglycerol acyltransferase/mycolyltransferase [*Mycobacterium leprae TN*]	NC_002677.1 (2418620..2419603)	fbpB

The purpose of this study was to evaluate the diagnostic value of three antigen-specific immune diagnostic tests, namely, the NDO-LID Rapid Test(antibody response), an antigen-specific enzyme linked immunosorbent assay (ELISA)(anti-LID-1, anti-NDO-LID, and anti-ND-O-BSA)(antibody response), and antigen-specific IFN-γ secretion in a whole blood assay (WBA) (stimulated by LID-1, ML89, ML2044 and ML2028)(antigen-specific CMI) for diagnosing leprosy in a hyperendemic area in China.

## Methods

### Ethics statement

This study was approved by the Medical Ethics Committee of Beijing Friendship Hospital, Capital Medical University, Beijing, P.R. China. Written informed consent was obtained from all adult participants, and all parents or guardians of child participants provided informed consent on their behalf. All of the procedures in the study involving human participants were performed in accordance with the ethical standards of the institutional and/or national research committee and with the 1964 Helsinki Declaration and its later amendments or comparable ethical standards.

### Study area and subjects

Eighty-three leprosy patients, who were referred to the Honghe Prefecture Skin Disease Prevention and Cure Institute in Honghe Autonomous Prefecture, Yunnan Province, were included in the study. Leprosy diagnosis was established based on clinical signs and symptoms, skin smears, skin biopsy, and neuro-physiologic examinations. The leprosy patients were classified into five groups based on the Ridley and Jopling[13] classification: tuberculoid (TT), borderline-tuberculoid (BT), borderline-borderline (BB), borderline-lepromatous (BL), and lepromatous (LL) groups. For data analysis in this study, leprosy patients were also classified into three groups: PB and MB, according to the WHO operational classification[14] during MDT, or post-MDT. One hundred and sixty-one controls from the same endemic region were included as non-leprosy controls. The controls were further classified into three groups: healthy household contacts (HHC), tuberculosis (TB), and endemic controls (EC).

### NDO-LID Rapid Test

Antigen-specific antibody detection by NDO-LID was performed as previously described[15]. Serum antibodies were measured by the NDO-LID rapid diagnostic test (RDT; procured from Orange Life, Rio de Janeiro, Brazil). Briefly, NDO-LID RDT was performed by first adding undiluted serum (10 μl) into the sample well within the test cassette, followed by the addition of running buffer (100 μl). Samples migrated through the cassette such that interactions with the test and/or control lines were revealed as red colored lines within the reading window. Tests were valid if the control line was observed. A positive result was defined by the presence of the test line. Visual results were interpreted after 20 minutes by two independent readers and scored subjectively as (±/+/++/+ + +), with faint (±) or no test line considered a negative result.

### Anti-LID-1, anti-NDO-LID and anti-ND-O-BSA by ELISA

ELISA microplate wells were coated overnight with the *M*. *leprae*-specific antigens LID-1 (1 μg/ml), NDO-LID (200 ng/ml) or synthetic PGL-I (200 ng/ml ND-O-BSA) in 0.1 M carbonate/bicarbonate coating buffer, pH 9.6 (50 μl). After 1 h in blocking buffer (1% bovine serum albumin in phosphate-buffered saline, pH 7.2, with 0.05% tween and 1% BSA/PBS/T), sera were diluted in blocking solution, tested at a 1:200 dilution (100 μl), and subsequently incubated for 2 h at room temperature (RT). Then, the wells were washed with PBS with 0.05% tween 20 (PBS/T, wash buffer) six times. Secondary peroxidase-conjugated anti-human IgM (anti-PGL-I), anti-human IgG (anti-LID-1) (1:20,000, Abcam, Cambridge, UK), or a combination of anti-human IgM and IgG antibodies (anti-NDO-LID) was added for another 2-h incubation period. Following this incubation, the wells were washed with PBS/T six times, followed by the addition of 100 μl of substrate (3,3’,5,5’-tetramethylbenzidine; TMB). After 15-minute incubation at RT, 50 μl of stop solution (H_2_SO_4_, 1 M) was added. Optical density (OD) values were determined with an ELISA plate reader (Asys Expert Plus-Microplate Reader UK) at 450 nm. The cut off for ELISA positivity was calculated from an OD value of 0.2, as described previously[15].

### IFN-γ release by *M*. *leprae*-specific antigen Stimulated WBA

WBA was performed as previously described. Briefly, undiluted, heparinized venous whole blood (Greiner) was collected. Whole blood was plated into 24-well plates (450 μl/well; Sigma, St. Louis, MO) within 2 h of collection and incubated with stimulants for 24 h at 37°C and 5% CO_2_. Each assay included stimulation with individual *M*. *leprae* recombinant proteins, including LID-1, ML89, ML2044, and ML2028 (provided by Dr. M.S. Duthie, Infectious Disease Research Institute (IDRI), Seattle, USA), at 100 μg/ml in PBS for experimental evaluations or 750 μg/ml PHA (Sigma) as a control treatment. Approximately 150 μl of plasma was collected and stored at -20°C until IFN-γ assessment. IFN-γ concentration was determined by ELISA according to the manufacturer’s instructions (U-CyTech Biosciences Human IFN-γ ELISA kit, CT201A, The Netherlands, CM). The IFN-γ ELISA employed had a detection limit of 2 pg/ml, and a threshold for positive responses was arbitrarily selected at 50pg/ml according to a previous study[15].

### Statistical analysis

Statistical analysis was performed primarily with GraphPad Prism software version 5.0 (GraphPad Software Inc., San Diego, CA, USA). The nonparametric Mann-Whitney U test was used to analyze differences between two groups. The Kruskal-Wallis test was used to analyze differences among three or more groups. Probability (p) values less than 0.05 were considered significant. The diagnostic utility of individual *M*. *leprae* antigen-specific responses for leprosy disease, including sensitivity, specificity, Youden’s index, and area under the receiver operator characteristic curve (AUC), were ascertained by receiver operator characteristics (ROC) curve analysis. The concordance between results was determined by kappa values (κ),and p values were calculated (Statistical Package for the Social Sciences (SPSS) version 16.0).

## Results

### Study area

The study was undertaken mainly in counties in Honghe Autonomous Prefecture, Yunnan (YN) Province, southwest China. Other cases were enrolled from the nearby autonomous prefectures of Chuxiong, Zhaotong and Kunming (provincial capital city) in YN. Honghe Autonomous Prefecture hadan estimated population of 4,470,000 in 2015 and is considered highly endemic for leprosy in China (annual new case detection rate of 1.13/100,000 from 2000–2007). According to data from the Honghe Prefecture Skin Disease Prevention and Cure Institute, 190 new cases were reported from 2010 to 2014[16].

### Basic characteristics of leprosy patients and controls

Eighty-three leprosy cases[MB, n = 38; PB, n = 23; and MDT, n = 22] and 161 controls [HHC, n = 119; TB, n = 11; and EC, n = 31] from the same endemic region were included. The basic information for each study group is summarized in Table 2.

**Table 2:** Clinical characteristics of the leprosy patients enrolled in this study.

Leprosy	Leprosy classification (n, %)		n	Gender ratio	Mean age	Bacterial index (BI)	
	WHO*	RJ**	(n, %)	(M/F)	Year (range)	Skin-slit smear	Pathology
	MB	LL	4	3/1	40.0 (24–59)	1–3.5+	5.5–6+
		BL	32	20/12	42.3 (21–91)	1–5+	2.2–5+
		BB	2	2/0	43.0 (34–52)	4+	2.6+
	PB	BT	14	7/7	46.0 (17–84)	0–1.2+	0–3.5+
		TT	9	7/2	44.9 (29–62)	0	0
	Post-MDT	LL	3	3/0	60.3 (54–68)	-	-
		BL	8	5/3	65.8 (48–80)	-	-
		BB	0	-	-	-	-
		BT	8	6/2	62.9 (52–80)	-	-
		TT	3	3/0	62.3 (42–78)	-	-
Controls	HHC		119	57/62	33.7 (2–87)	-	-
	TB -		11	8/3	44.5 (28–77)	-	-
	EC		31	18/13	39.2 (32–48)	-	-

n: number of patients, with percentages in parentheses.

*WHO: Operational classification proposed by the World Health Organization.

** RJ: Ridley-Jopling classification.

### Comparison of NDO-LID Rapid Test, *M*. *leprae* antigen-specific ELISA and IFN-γ in WBA by positive responses

Serum samples were evaluated by the NDO-LID Rapid Test, *M*. *leprae* antigen-specific ELISA, and *M*. *leprae* antigen-specific secretion of IFN-γ in WBA based on the positive response rate (Table 3).

**Table 3:** Comparison of NDO-LID Rapid Test, *M*. *leprae* antigen-specific ELISA and WBA positive response rates.

Leprosy classification		Rapid Test	ELISA (OD)				IFN-γ Secretion by WBA (pg/ml)			
		NDO-LID	LID-1	NDO-LID	ND-O-BSA		LID-1	ML89	ML2044	ML2028
	Cut off^α^	-	0.2	0.2	0.2	Cut off^β^	50	50	50	50
	Total (n)	n (%)	n (%)	n (%)	n (%)	Total (n)	n (%)	n (%)	n (%)	n (%)
MB	38	36 (94.7%)	35(92.1%)	35(92.1%)	34(89.5%)	35	14/35 (40%)	10/35 (28.6%)	11/35 (31.4%)	11/35 (31.4%)
PB	23	15 (65.2%)	12(52.2%)	11(47.8%)	14(60.9%)	23	13 (56.5%)	11 (47.8%)	14 (60.7%)	11 (47.8%)
Post-MDT	22	7 (31.8%)	9(45.0%)	9(45.0%)	11(55.0%)	20	12/20 (54.5%)	14/20 (63.6%)	7/20 (31.8%)	9/20 (40.9%)
HHC	119	34 (28.6%)	53(44.5%)	31(26.0%)	63(52.9%)	116	56/116 (48.3%)	51/116 (44.0%)	49/115 (42.6%)	40/116 (34.5%)
TB	11	1 (9.1%)	2(18.2%)	0	2(18.2%)	11	0	0	0	0
EC	31	10 (33.3%)	4(12.9%)	5(16.1%)	12(38.7%)	31	16 (51.6%)	15 (48.4%)	13 (41.9%)	8 (25.8%)

α: Cut off value of *M*. *leprae-*specific antigen ELISA was defined as 0.2 OD value.

β: Cut off value of IFN-γ was defined as 50 pg/ml.

For the NDO-LID Rapid Test, the positive response rates were higher in the MB than in the PB group[MB *vs* PB: 94.7% (36/38) *vs* 65.2% (15/23)]. For *M*. *leprae* antigen-specific ELISA, a trend similar to that observed for the NDO-LID Rapid Test was noted: the positive response rates were also higher in the MB than in the PB group[MB *vs* PB: 92.1% (35/38) *vs* 52.2% (12/23) against LID-1, 92.1%(35/38) *vs* 47.8%(11/23) against NDO-LID, and 89.5% (34/38) *vs* 60.9% (14/23) against ND-O-BSA]. Both methods also demonstrated higher response rates in the MB group than in the post-MDT, HHC, EC, and TB groups.

For WBA, however, the positive response rates were higher in the PB group than in the MB group[MB:PB: 40%(14/35) *vs* 56.5% (13/23)for LID-1, 28.6%(10/35) *vs* 47.8%(11/23) for ML89, 31.4%(11/35) *vs* 60.7%(14/23) for ML2044, and 31.4%(11/35) *vs* 47.8%(11/23) for ML2028]. WBA also showed higher response rates in the PB group than in the post-MDT, HHC, EC, and TB groups, except for the ML89 antigen in the post-MDT and EC groups.

When the same samples were evaluated using the NDO-LID Rapid Test, confirmation was achieved in 94.7%(36/38) of MB patients, and a high degree of agreement was observed between LID-1(92.1%), NDO-LID (92.1%), and ND-O-BSA (89.5%) ELISA. For PB patients, the NDO-LID Rapid Test reached 65.2% confirmation, which was slightly higher than the results obtained for LID-1(52.2%), NDO-LID(47.8%), and ND-O-BSA (60.9%) ELISA (Table 2). However, the NDO-LID Rapid Test showed positive responses in 33.3% (10/31) of the EC group, which was similar to the rate for ND-O-BSA(38.7%, 12/31) but higher than those for LID-1(12.9%, 4/31) and NDO-LID(16.1%, 5/31) (Table 3).This finding indicates that the NDO-LID Rapid Test is more sensitive than *M*. *leprae*-specific antigen ELISA (anti-LID-1 and anti-NDO-LID) for detecting leprosy patients, especially MB patients, but has reduced specificity.

### Comparing the consistency of NDO-LID Rapid Test, *M*. *leprae* antigen-specific ELISA and WBA by kappa test

A kappa test analyzes for the agreement of results collected from various test formats. When a kappa test was performed between the NDO-LID Rapid Test and *M*. *leprae* antigen-specific ELISA and between the NDO-LID Rapid Test and WBA, good agreement was only observed between the NDO-LID Rapid Testand *M*. *leprae* antigen-specific ELISA (anti-LID-1, anti-NDO-LID, and anti-ND-O-BSA), with indexes of 0.868, 0.868 and 0.842, respectively (p values of 0.000, 0.000, and 0.000, respectively) for the MB group(Table 4). This finding indicates that the two tests showed high consistency for the diagnosis of MB leprosy patients.

**Table 4:** Comparison of NDO-LID Rapid Test, *M*. *leprae* antigen-specific ELISA and WBA by kappa test.

**NDO-LID Rapid Test *vs***	**ELISA**	**Kappa test**		**WBA**	**Kappa test**	
		Kappa	p value		Kappa	p value
**MB**	LID-1	0.8680	0.0000	LID-1	0.2860	0.0070
	NDO-LID	0.8680	0.0000	ML89	0.2390	0.0070
	ND-O-BSA	0.8420	0.0000	ML2044	0.2680	0.0040
				ML2028	0.2680	0.0040
**PB**	**ELISA**	**Kappa test**		**WBA**	**Kappa test**	
		Kappa	p value		Kappa	p value
	LID-1	0.1740	0.2340	LID-1	0.2170	0.1390
	NDO-LID	0.1300	0.3690	ML89	0.1300	0.3690
	ND-O-BSA	0.2610	0.0770	ML2044	0.2610	0.0770
				ML2028	0.1300	0.3690
**Post-MDT**	**ELISA**	**Kappa test**		**WBA**	**Kappa test**	
		Kappa	p value		Kappa	p value
	LID-1	-0.2730	0.0690	LID-1	0.8100	0.5800
	NDO-LID	-0.2730	0.0690	ML89	0.1800	0.8990
	ND-O-BSA	-0.1820	0.2200	ML2044	-0.3300	0.0320
				ML2028	-0.2300	0.1290

### Discriminating between leprosy patients and controls with *M*. *leprae* antigen-specific ELISA

**Fig 1:**
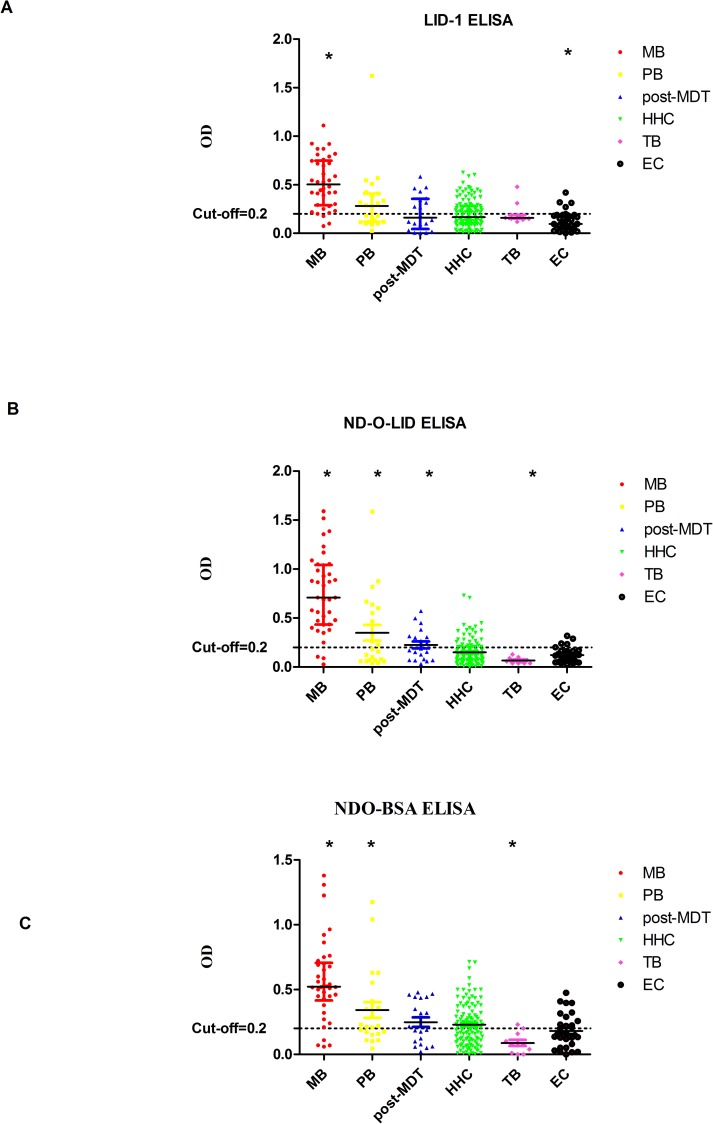
Scatter-dot plots of OD detected in *M*. *leprae* proteins by ELISA. Differences between analyte levels were evaluated by the Mann Whitney U test for non-parametric data analysis. Representative plots show the analyte OD in the LID-1, NDO-LID, and ND-O-BSA ELISA for participants with leprosy (MB, PB and post-MDT) and without leprosy disease controls(HHC, TB and EC). (A)* = p<0.05 for LID-1(MB *vs* PB, post-MDT, HHC, TB and EC; EC *vs* PB, HHC and TB);(B) * = p<0.05 for NDO-LID (MB *vs* PB, post-MDT, HHC, TB and EC; PB *vs* HHC, TB and EC; post-MDT *vs* HHC, TB and EC; TB *vs* HHC and EC); (C)* = p<0.05 for ND-O-BSA (MB *vs* PB, post-MDT, HHC, TB and EC; PB *vs* TB and EC; TB *vs* post-MDT, HHC and EC). Bars in the scatter-dot plots represent the median plus interquartile range of analyte concentrations. *M*. *leprae* Ag: LID-1, ML89, ML2044, ML2028; IFN-γ: Interferon gamma.

For all three *M*. *leprae* antigens (LID-1, NDO-LID and ND-O-BSA ELISA), the OD values showed significant differences for MB *vs* the PB, post-MDT, HHC, TB or EC groups, and PB *vs* EC(Fig 1). Of note, NDO-LID was better than the other two antigens (LID-1 and ND-O-BSA) at discriminating PB leprosy patients from non-leprosy controls (Table 5). In addition, we evaluated the diagnostic ability of *M*. *leprae* antigen-specific ELISA using ROC curve analysis, AUC, sensitivity and specificity (Table 6) and demonstrated that this method had a relatively high specificity but low sensitivity.

**Table 5:** P values of *M*. *leprae* antigen-specific ELISA between the leprosy group and control groupsfrom the Kruskal-Wallis test(among three groups) and the Mann-Whitney U test(between two groups).

*M*. *leprae* antigens	Leprosy classification	Total	PB	post-MDT	HHC	TB	EC
	MB	<0.0001	0.0007	<0.0001	<0.0001	0.0001	<0.0001
	PB			0.2280	0.1041	0.6321	0.002
	post-MDT				0.8336	0.5773	0.1800
	HHC					0.6818	0.005
	TB						0.0478
NDO-LID	MB	<0.0001	0.0001	<0.0001	<0.0001	<0.0001	<0.0001
	PB			0.6523	0.0378	0.0037	0.0373
	post-MDT				0.0331	0.0024	0.0191
	HHC					0.0091	0.4935
	TB						0.0265
ND-O-BSA	MB	<0.0001	0.0022	<0.0001	<0.0001	<0.0001	<0.0001
	PB			0.4216	0.1408	0.0005	0.0209
	post-MDT				0.6462	0.0111	0.1619
	HHC					0.0018	0.1489
	TB						0.0319

**Table 6:** ROC of the diagnostic potential of OD detected in *M*. *leprae* antigen-specific ELISA for leprosy diagnosis.

*M*. *leprae* Antigens	Subgroups of Cases		p value	AUC	Sensitivity (%)	Specificity (%)	Youden's Index
LID-1	MB *vs*	PB	0.0007	0.7603	39.13%	94.74%	0.3387
		post MDT	<0.0001	0.823	35.00%	97.37%	0.3237
		HHC	<0.0001	0.8505	50.42%	94.74%	0.4516
		TB	0.0001	0.8804	54.55%	94.74%	0.4929
		EC	<0.0001	0.9334	51.61%	97.37%	0.4898
	PB *vs*	EC	0.0020	0.7482	41.94%	95.65%	0.3759
	HHC *vs*	EC	0.0050	0.6641	6.46%	99.16%	0.0562
	TB *vs*	EC	0.0468	0.7038	58.06%	90.91%	0.4897
NDO-LID	MB *vs*	PB	0.0001	0.7929	34.78%	97.37%	0.3215
		post MDT	<0.0001	0.8895	30.00%	97.37%	0.2737
		HHC	<0.0001	0.9285	38.66%	97.37%	0.3603
		TB	<0.0001	0.9653	72.73%	97.37%	0.7010
		EC	<0.0001	0.9423	38.71%	97.37%	0.3608
	PB *vs*	HHC	0.0377	0.6372	13.45%	91.30%	0.0475
		TB	0.0037	0.8123	36.36%	95.65%	0.3201
		EC	0.0366	0.6676	29.03%	91.30%	0.2033
	post-MDT *vs*	HHC	0.0329	0.6494	20.17%	95.00%	0.1517
		TB	0.0023	0.8364	36.36%	95.00%	0.3136
		EC	0.0186	0.6968	29.03%	95.00%	0.2403
	HHC *vs*	TB	0.0091	0.7383	36.36%	86.55%	0.2291
	TB *vs*	EC	0.0257	0.7287	58.06%	90.91%	0.4897
ND-O-BSA	MB *vs*	PB	0.0022	0.7357	56.52%	86.84%	0.4336
		post MDT	<0.0001	0.8434	20.00%	97.37%	0.1737
		HHC	<0.0001	0.8430	15.97%	97.37%	0.1334
		TB	<0.0001	0.9414	36.36%	97.37%	0.3373
		EC	<0.0001	0.8862	22.58%	97.37%	0.1995
	PB *vs*	TB	0.0005	0.8755	63.64%	95.65%	0.5929
		EC	0.0205	0.6858	25.81%	95.65%	0.2146
	post-MDT *vs*	TB	0.0105	0.7818	36.36%	95.00%	0.3136
	HHC *vs*	TB	0.0017	0.7861	18.18%	99.16%	0.1734
	TB *vs*	EC	0.0308	0.7214	38.71%	90.91%	0.2962

All of the analytes that showed significant differences (p<0.05) between leprosy and non-leprosy controls according to receiver operating characteristic (ROC) analysis are shown. Sensitivity and specificity were selected based on Youden’s index. AUC = Area under the receiver operator characteristic curve.

### Evaluation of IFN-γ as a potential diagnostic host biomarker for leprosy

**Fig 2:**
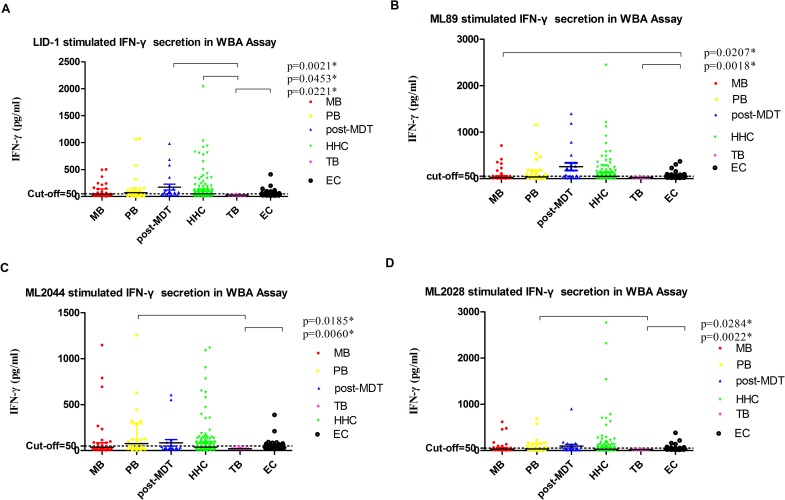
Scatter-dot plots of host IFN-γ detected in antigen-specific overnight WBA supernatants. Differences in analyte levels were evaluated by the Mann Whitney U test for non-parametric data analysis. Representative plots show the levels of analytes in the overnight whole blood culture supernatants of participants with leprosy (MB, PB and post-MDT) and without leprosy controls(HHC, TB and EC). Bars in the scatter-dot plots represent the median plus interquartile range of the analyte concentration. *M*.*leprae* Ag: LID-1(A), ML89(B), ML2044(C), ML2028(D); IFN-γ: Interferon gamma.

**Fig 3:**
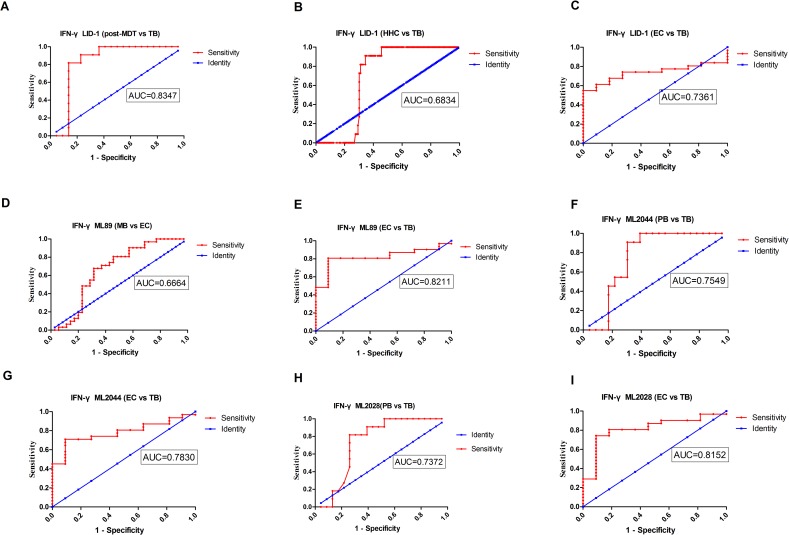
ROC curves of host marker IFN-γ detected in stimulated overnight WBA supernatants. Representative ROC curves showing the accuracy of IFN-γ as a marker discriminating between participants with leprosy (MB, PB and post-MDT) and controls without leprosy disease (HHC, TB and EC). All of the markers had AUC≥0.70 except IFN-γ LID-1 (HHC *vs* TB), and IFN-γ ML89 (MB *vs* EC). *M*. *leprae* Ag: (A) post-MDT, (B) HHC, and (C) EC *vs* TB stimulated by LID-1;(D) MB and (E) TB *vs* EC stimulated by ML89;(F) PB and (G) EC *vs* TB stimulated by ML2044;(H) PB and (I) EC *vs* TB stimulated by ML2028. IFN-γ: Interferon gamma.

We compared the analyte levels detected in *M*. *leprae* antigen-stimulated WBA supernatants in leprosy patients with the levels obtained from the non-leprosy control groups using the mean and standard deviation(SD)(Fig 2) and the median and range(Table 7). As described previously, newly diagnosed PB patients produce more IFN-γ than MB patients. We also evaluated the diagnostic potential of IFN-γ by ROC curve analysis and AUC. IFN-γ levels were significantly different in (1) MB *vs* EC when stimulated with ML89(AUC = 0.6664); (2) PB *vs* TB when stimulated with ML2044 and ML2028(AUC = 0.7549 and 0.7372, respectively); (3) post-MDT *vs* TB when stimulated with LID-1(AUC = 0.8347); (4) HHC *vs* TB when stimulated with LID-1(AUC = 0.6834); and (5) EC *vs* TB when stimulated with LID-1, ML89, ML2044 and ML2028(AUC = 0.8211, 0.8152, 0.7830, and 0.7361, respectively)(Fig 3, Table 8).

**Table 7:** Median and range values of IFN-γ as a potential host biomarker detected in overnight culture supernatant by WBA for the leprosy group and control groups.

Marker/*M*.*leprae* Antigens		MB	PB	post-MDT	HHC	EC	TB
IFN-γ/LID-1	Median	44.56	68.76	73.51	49.04	25.9	53.46
	Minimum	0.93	1.78	14.27	1.12	22.18	14.41
	Maximum	502.7	1079	981.3	2036	46.17	411.9
IFN-γ/ML89	Median	23.7	36.72	60.95	48.36	22.18	49.33
	Minimum	0.75	1.29	10.76	0.89	17.31	14.38
	Maximum	710.4	1155	1397	2441	51.63	371.4
IFN-γ/ML2044	Median	37.33	76.14	29.57	38.26	20.38	35.3
	Minimum	0.78	1.78	10.45	0.89	16.45	16.05
	Maximum	1150	1257	606.9	1118	42.44	388
IFN-γ/ML2028	Median	29.73	33.19	25.75	26.83	18.61	32.5
	Minimum	0.75	1.29	10.45	0.85	14.35	14.15
	Maximum	620.1	685.1	900.2	2758	47.79	380.6

Median levels of analytes (pg/ml) and ranges (minimum and maximum) showing accuracies at discriminating between leprosy and controls in overnight culture supernatants for all of the study participants.

**Table 8:** ROC of the diagnostic potential of IFN-γ detected in overnight culture supernatant for leprosy.

Subgroups of Cases	IFN-γ/*M*.*leprae* Antigens	p value	AUC	Sensitivity (%)	Specificity (%)	Youden's Index
MB vs EC	IFN-γ/ML89	0.0204	0.6664	67.74%	68.57%	0.3631
PB vs TB	IFN-γ/ML2044	0.0176	0.7549	90.91%	69.57%	0.6048
	IFN-γ/ML2028	0.0272	0.7372	81.82%	73.91%	0.5573
post-MDT vs TB	IFN-γ/LID-1	0.0020	0.8347	81.82%	86.36%	0.6818
HHC vs TB	IFN-γ/LID-1	0.0449	0.6834	81.82%	68.97%	0.5079
EC vs TB	IFN-γ/ML89	0.0017	0.8211	80.65%	90.91%	0.7156
	IFN-γ/ML2028	0.0021	0.8152	74.19%	90.91%	0.651
	IFN-γ/ML2044	0.0058	0.783	70.97%	90.91%	0.6188
	IFN-γ/LID-1	0.0213	0.7361	61.29%	90.91%	0.522

All of the analytes that showed significant differences (p<0.05) between leprosy and uninfected controls according to ROC analysis are shown. Sensitivity and specificity were selected based on Youden’s index. AUC = Area under the receiver operator characteristics curve.

## Discussion

Widespread application of MDT therapy has led to major advances in leprosy control, with sharp declines in prevalence rates in the vast majority of countries over the last 20 years[15,17]. However, the disease remains a public health concern in many regions. In 2010, China reported 1324 new cases of leprosy to the WHO[18]. The majority of cases in China came from the ethnically diverse, mountainous, and underdeveloped southwest provinces of Yunnan, Guizhou, and Sichuan[19,20]. Honghe Autonomous Prefecture inYunan is considered a highly endemic area for leprosy in China. We enrolled 83 leprosy patients and 161 controls from this endemic region in this study to evaluate the ability of several diagnostic tests to correctly diagnose different categories of leprosy patients. We found that the NDO-LID Rapid Test and *M*. *leprae* antigen-specific ELISA was useful to diagnose leprosy patients in hyperendemic areas of leprosy disease, especially MB patients. The former method provides a point-of-care measurement of antibodies and had higher sensitivity but lower specificity than the latter. *M*. *leprae* antigen-specific IFN-γ secretion in WBA has diagnostic value for distinguishing PB from TB but not for distinguishing PB and HHC or EC.

*M*. *leprae-*specific antigen tests have been developed as useful tools to diagnose leprosy. LID-1, NDO-LID, and ND-O-BSA(also named PGL-I), as representative *M*. *leprae*-specific antigens, have been widely evaluated as leprosy diagnostics in the hyper-endemic regions of Brazil[20–23], Colombia, the Philippines[24], and China[15] and have been demonstrated to be excellent tools for detecting MB leprosy patients in a simple and highly quantitative manner[24], predicting patients susceptible to developing leprosy type 2 reactions (T2R)[23], and distinguishing leprosy from other confounding dermatoses[18].

The NDO-LID Rapid Testwas compared with *M*. *leprae* antigen-specific ELISA and demonstrated a high degree of sensitivity but significant differences in specificity for leprosy diagnosis[25]. Therefore, this test is an effective tool for screening and identifying individuals at high risk who might benefit from regular monitoring[26]. Our previous study showed that confirmation was achieved in 95% of MB leprosy patients with the NDO-LID Rapid Test, and a high degree of agreement was observed with LID-1, NDO-LID, and ND-O-BSA ELISA[15]. In addition, 63.6% of PB leprosy patients were confirmed, and the NDO-LID Rapid Test had a higher detection rate in PB leprosy patients than LID-1, ND-O-BSA, and NDO-LID ELISA[15]. In this study, we enlarged the sample size and obtained results similar to those of previous studies. These data indicate an improved capacity of the NDO-LID Rapid Test over *M*. *leprae* ELISA for detecting the disease. However, the test also suffers from higher positive responses in the EC group than did NDO-LID and LID-1 ELISA. This implies that the NDO-LID Rapid Test was more sensitive but less specific than *M*. *leprae* antigen-specific ELISA(anti-LID-1 and anti-NDO-LID) for discriminating the leprosy patient group fromthen on-leprosy EC group. Despite the relatively low specificity, the NDO-LID Rapid Test, as a low-tech, robust assay, can still be applied in resource-poor settings to measure the immune response to *M*. *leprae* infection and can be used as a tool for leprosy screening in combination with good specificity confirmation tests, which will lead to timely treatment and reduced transmission[27].

We also evaluated the capacity of *M*. *leprae* antigen-specific ELISA to discriminate between the leprosy and control groups. All three *M*. *leprae* antigens (LID-1, NDO-LID and ND-O-BSA) were able to discriminate the MB group from all other leprosy and non-leprosy groups and the PB leprosy group from the non-leprosy EC groups, whereas only NDO-LID was able to discriminate the PB leprosy group from the non-leprosy HHC group. This indicates that all three *M*. *leprae* antigens have potential and specific value for research and medical applications. As described before, the *M*. *leprae* antigen-specific ELISA had lower sensitivity but better specificity than the NDO-LID Rapid Test. ELISA detection of specific antibodies may be preferred for confirming diagnoses, differentiating leprosy from other dermatological conditions, and performing follow-up studies for leprosy HHC and indeterminate leprosy, which are very early signs of the disease that are often missed by family members and medical personnel in the endemic area[27].

Cytokines such as IFN-γ have recently been studied as diagnostic host biomarkers for leprosy. *M*. *leprae-*specific antigens, such as *M*. *Leprae* crude antigens (*M*. *leprae* cell sonicate, MLCS), *M*. *leprae* recombinant protein (rML)(LID-1), *M*. *leprae* diffusion proteins[46f(ML0405+ML0568) and 73f(ML2028+ML2346+ML2044)] and combinations of rML (46f+LID-1, ML0276+LID-1, ML2055+ML1632+ML2044, ML0276+46f, and ML2055+LID-1) were used in these studies[20,28–31]. IFN-γ and CXCL10 were evaluated as potential diagnostic host markers for PB leprosy patients in the hyper-endemic regions of Brazil[20,28–31] and China[15]. Newly diagnosed PB patients produced more IFN-γ than MB patients[28–31], and IFN-γ was helpful in the differential diagnosis of leprosy from other confounding dermatoses[2]. CXCL10 discriminated PB from EC only in ML0276+LID-1 WBA; however, CXCL10 could not discriminate active disease (PB) from HHC individuals[28]. In this study, we also demonstrated that for new cases, PB patients produced more IFN-γ than MB patients; however, IFN-γ did not discriminate active disease (PB) from HHC or EC individuals.

In this study, we investigated the accuracy of IFN-γ as a host marker detected in supernatants after stimulation of whole blood with *M*. *leprae-*specific antigens (LID-1, ML89, ML2044, and ML2028) in an overnight culture assay and compared the IFN-γ marker levels in leprosy and non-leprosy control groups. Although IFN-γ can be useful as a host biomarker that contributes to a diagnostic signature of MB *vs* EC and that distinguishes PB *vs* TB groups, there was no evidence that IFN-γ was able to discriminate between the PB and HHC or EC groups. To screen novel *M*. *leprae-*specific antigens, combining different *M*. *leprae* antigens and facilitating a multi-cytokine analyte model may achieve improved diagnostic potential.

This study is limited by the small sample size, especially in the PB group. Antigen-specific immune responses have had limited diagnostic ability for leprosy disease and until recently have only been used for seroepidemiological investigation in hyperendemic areas of leprosy disease or in patients clinically suspected of having leprosy disease. However, the results should be interpreted with caution. Only a very limited number of *M*. *leprae-*specific antigens(LID-1, ML89, ML2044, and ML2028) and only one potential diagnostic host biomarker (IFN-γ) were tested for leprosy diagnosis in this study. Future studies should focus on additional *M*. *leprae*-specific antigens as well as additional host biomarkers.

In conclusion, the NDO-LID Rapid Test and *M*. *leprae* antigen-specific ELISA were helpful for diagnosing leprosy in hyperendemic areas of leprosy disease, especially for MB patients. The former had higher sensitivity but lower specificity than the latter. Although IFN-γ has been widely studied as a potential biomarker for PB leprosy patients, more research is needed to identify feasible *M*. *leprae-*specific antigens and other appropriate host biomarkers to improve its diagnostic value in PB patients in future studies.
